# Peak ${\dot{V}}_{O_{2}Q}$: A new approach for the interpretation of cardiorespiratory fitness estimates

**DOI:** 10.1113/EP093195

**Published:** 2025-10-31

**Authors:** Ben Knox‐Brown, Joshua Barnes, Chris Harding, Jonathan Fuld, Karl P. Sylvester

**Affiliations:** ^1^ Respiratory physiology, Cambridge University Hospitals, NHS Foundation Trust Cambridge UK; ^2^ Respiratory physiology, Royal Papworth Hospital NHS Foundation Trust Cambridge UK; ^3^ National Heart and Lung Institute, Imperial College London London UK

**Keywords:** exercise physiology, mortality, normative values, patients

## Abstract

There are difficulties with the standardisation of interpretative strategies for peak oxygen uptake (peak ${\dot{V}}_{O_{2}}$) related to the quality of reference equations. We aimed to investigate the utility of peak ${\dot{V}}_{O_{2}Q}$, a novel method reflecting how far a measured value is from the 1st percentile. We retrospectively analysed data from patients referred for a cardiopulmonary exercise test (CPET) at Cambridge University Hospital (CUH) and Royal Papworth Hospital (RPH). Data were included from those 18 years or older. We investigated the stability of the 1st percentile overall, then stratified by sex and age group. We calculated the peak ${\dot{V}}_{O_{2}Q}$ (measured peak ${\dot{V}}_{O_{2}}$/1st percentile) and investigated its association with all‐cause mortality using Cox regression analysis. Data from 1377 patients were included in the analyses: 590 from CUH (mean age 55.5 (15.4) years, 47% female) and 787 from RPH (mean age 46.1 (15.3) years, 48% female). The 1st percentile value for peak ${\dot{V}}_{O_{2}}$ was 9.5 mL kg^−1^ min^−1^ (95% CI: 8.8–9.9) and was consistent across sex and age groups. The mean peak ${\dot{V}}_{O_{2}Q}$ was 2.3 (0.8); it declined with age and was lowest in patients referred for heart transplant. Data on all‐cause mortality were available for all patients from CUH. Median follow‐up time was 3.8 (2.2–9.6) years, during which time 96 of 590 (16%) patients died. A 1‐unit increase in peak ${\dot{V}}_{O_{2}Q}$ was associated with a 60% reduction in risk of all‐cause mortality. We propose the peak ${\dot{V}}_{O_{2}Q}$ as an alternative means to interpret cardiorespiratory fitness estimates that does not require reference equations.

## Abstract (highlights)

1. **What is the central question of this study?**
  Can a 1st percentile (minimally survivable) value for peak ${\dot{V}}_{O_{2}}$ be used as a reference standard against which all people are compared?
2. **What is the main finding and its importance**
  In two hospital‐based cohorts, the 1st percentile value for peak ${\dot{V}}_{O_{2}}$ was 9.5 mL kg^−1^ min^−1^. The derived quotient (measured peak ${\dot{V}}_{O_{2}}$/1st percentile) declined with age and was lowest in the most unwell patients. A 1‐unit increase in peak ${\dot{V}}_{O_{2}Q}$ (greater distance from minimum survivable value) was associated with a 60% reduction in risk of all‐cause mortality.

## 1 INTRODUCTION

Maximum oxygen consumption (${\dot{V}}_{O_{2}\max}$) is the gold standard measure of cardiorespiratory fitness; it is assessed by a cardiopulmonary exercise test (CPET). Clinically (usually referred to as peak ${\dot{V}}_{O_{2}}$), it is a measure of functional capacity (Cooper & Storer, 2001), used for pre‐operative risk stratification (Lindenmann et al., 2020; Swank et al., 2012), and to investigate causes of unexplained breathlessness (Huang et al., 2020). At population level, it is one of the strongest metrics associated with morbidity and mortality across a range of diseases (Cai et al., 2023; Gonzales et al., 2021; Steell et al., 2019).

The ability to predict incident morbidity and mortality is important in both clinical and general populations. It allows for the early identification of those at risk, enabling the implementation of preventative strategies, which include both lifestyle modification and pharmacological therapy (Choi & Rhee, 2020; Oh et al., 2023). Early disease intervention has been shown to have both a cost and quality of life benefit (Oude Wolcherink et al., 2023; Sediqzadah et al., 2022). Despite its wide use, there are difficulties related to the standardisation of interpretative strategies for peak ${\dot{V}}_{O_{2}}$, which limit its utility. The primary issue is the quality of reference equations and whether they can be trusted to differentiate between a normal versus abnormal response to exercise (Rapp et al., 2018). The most widely used reference equations in adults are those proposed by Koch et al. (2009), using a general population sample from Pomerania, Germany. These equations were derived from just 534 individuals, with few individuals over the age of 65 years. Similarly, another set of equations, developed by Wasserman et al. (1987), are over‐represented by middle age, normal weight males. More recently, data from the Fitness Registry and the Importance of Exercise National Database (FRIEND) has been used to derive reference equations for peak ${\dot{V}}_{O_{2}}$ (Myers et al., 2017). Despite the large sample size, these equations provide minimal improvement in the average error between measured and predicted peak ${\dot{V}}_{O_{2}}$, nor do they dramatically improve prediction of clinical outcomes (Myers et al., 2021).

Due to the wide variation in normal standards for peak ${\dot{V}}_{O_{2}}$, a simplified method for its interpretation is needed. One such approach would be to generate a physiological quotient, which compares a measured value to a lower boundary (minimal survivable value). This approach has previously been demonstrated for the spirometry parameter forced expiratory volume in 1 s (FEV_1_), where the 1st percentile for FEV_1_ was found to be different in males and females but stable across age. The measured FEV_1_ was divided by the 1st percentile value, giving the FEV_1_Q, a measure of how many turnovers an individual's FEV_1_ is from the 1st percentile. Interestingly, the FEV_1_Q was shown to improve the association with mortality and allow for much simplified interpretation compared to other derivatives of FEV_1_, including percent predicted and *z*‐score (Balasubramanian et al., 2024; Miller & Pedersen, 2010).

We hypothesised that as peak ${\dot{V}}_{O_{2}}$ measured in mL kg^−1^ min^−1^ accounts for body weight, unlike with FEV_1_, it would be possible to derive a universal 1st percentile value independent of sex. To investigate this, we used patient data from two hospitals to explore the feasibility of generating a physiological quotient for peak ${\dot{V}}_{O_{2}}$, and its subsequent association with all‐cause mortality.

## 2 METHODS

### 2.1 Study population

We retrospectively analysed data from patients referred for CPET at Cambridge University Hospital (CUH) National Health Service Foundation Trust (NHS FT) and Royal Papworth Hospital NHS FT (RPH), for any referral reason. Data were collected from CPETs conducted between the 1 January 2012 and 31 December 2024 at CUH, and 1 January 2021 and the 31 December 2024 at RPH. Data were included in this study if patients were 18 years or older at the time of testing and performed a maximal exercise test, defined as cardiovascular limitation (heart rate ≥90% of predicted or a heart rate reserve (HRR) ≤15 bpm), or ventilatory limitation (breathing reserve less than 15 L min^−1^ or 15% of maximum predicted ventilation) (Pritchard et al., 2021). The rational for these criteria is that to accurately quantify a 1st percentile value, the test should be physiologically maximal and not impacted by subjective criteria such as perceived symptoms at peak exercise. This could falsely reduce the 1st percentile, due to the inclusion of submaximal exercise data. For individuals with serial CPET measurements, the first maximal measurement was included in the analyses. As this was a retrospective analysis of routinely collected healthcare data, individual patient consent was not required. Approval to use the data from CUH was provided by the EHR Research and Innovation (ERIN) database data access committee, reference: A097236, IRAS: 318784. Approval to use the data from RPH was provided by the Papworth Cardiorespiratory Physiology Research Database (PCRPRD) data access committee, reference PCRPRD0002, IRAS: 346834. Research was conducted according to the ethical principles of the *Declaration of Helsinki*.

### 2.2 Data collection

CPETs were performed as part of routine care and were conducted according to the Association for Respiratory Technology and Physiology (ARTP) standards (Pritchard et al., 2021). Prior to the CPET, the work rate protocol (W min^−1^) was calculated according to height, weight, age and sex, using equations from Cooper & Storer (2001). At both sites, CPETs were performed on a ramp‐incremental cycle ergometer (Ergoline, Bitz, Germany). At CUH, prior to 2016 the Jaeger Oxycon Pro Metabolic Cart was used for measurement, and from 2016 the Vyntus CPX Metabolic Cart (Jaeger, GMBH) was used. At the time, appropriate physiological and biological controls were implemented to ensure continuity. Furthermore, the systems have been shown to have equal agreement between ventilation and gas exchange metrics (Groepenhoff et al., 2017). 355 of 590 (60%) patients at CUH were tested on the Oxycon Pro. The Vyntus™ CPX Metabolic Cart (Jaeger, GMBH) was used in all patients at RPH. Patients first performed spirometry to American Thoracic Society/European Respiratory Society standards (Graham et al., 2019), to assess lung function and to determine maximal voluntary ventilation (MVV = FEV_1_ × 40). Patients were then fitted with an ECG monitor, blood pressure cuff, mask and pulse oximeter. Once on the bike, the metabolic cart was connected to the mask via a digital volume transducer adapter to allow breath by breath analysis. Initially, resting cardiovascular, respiratory and metabolic measurements were collected. This was followed by 3 min of unloaded pedalling and approximately 8–12 min of loaded cycling. Patients were instructed to keep their pedalling cadence at 60 rpm until they could no longer keep going due to exhaustion or the premature termination criteria was met (Pritchard et al., 2021). Patients then observed a recovery period. Anaerobic threshold was determined using the V‐slope method. Peak ${\dot{V}}_{O_{2}}$ was taken from the highest 30 s average of ${\dot{V}}_{O_{2}}$ during the test. CPET parameters were reported as raw values and as a percent of predicted using reference equations from Koch et al. (2009).

In addition to CPET parameters, age, sex, height, weight and referral reason were extracted from the CPET databases. Data on all‐cause mortality was available only for patients who underwent CPET at CUH. For those patients who were deceased, time from CPET to death was extracted from the medical record. For all other patients, time between CPET and 31 December 2024 was calculated.

### 2.3 Outcome measures

The primary outcomes were the stability of the 1st percentile for peak ${\dot{V}}_{O_{2}}$ in mL kg^−1^ min^−1^ across sex and age groups, and the subsequent calculation of the peak ${\dot{V}}_{O_{2}Q}$, which represents the number of turnovers a given peak ${\dot{V}}_{O_{2}}$ measurement is from the 1st percentile value, calculated as:

$$\mathrm{Peak}\;{\dot{V}}_{O_{2}Q}=\mathrm{measured}\;\mathrm{peak}\;{\dot{V}}_{O_{2}}/1\mathrm{st}\;\mathrm{percentile}\;\mathrm{for}\;\mathrm{peak}\;{\dot{V}}_{O_{2}}$$

The secondary outcome was the association of the peak ${\dot{V}}_{O_{2}Q}$ with survival in comparison to the peak ${\dot{V}}_{O_{2}}$ expressed in mL kg^−1^ min^−1^, as percent predicted, and according to Weber functional class (Weber et al., 1982).

### 2.4 Statistics

Normality of the data was determined through inspection of Q–Q plots and histograms. For normally distributed data, the mean and standard deviation (SD) were reported, for non‐normal data, median and interquartile range (IQR) were reported. Categorical variables were summarised as the number and percentage in each group. To derive the 1st percentile value for peak ${\dot{V}}_{O_{2}}$ mL kg^−1^ min^−1^, we combined the databases at CUH and RPH. The rationale for this was two‐fold, firstly, as both hospitals serve the East of England but have different patient cohorts, it allowed for the creation of a more representative database. Secondly, the combined database increased the sample size for us to explore the variation in the 1st percentile for peak ${\dot{V}}_{O_{2}}$ in stratified analyses. We investigated the stability of the 1st percentile, by calculating 1st percentile values with 95% confidence intervals (95% CI) overall, then stratified by sex (male/female), and by age group (<40, 40–49, 50–59, 60–69 and ≥70 years). We plotted results graphically and decided on a consensus 1st percentile value that was representative of any variation seen across sex and age. We calculated the peak ${\dot{V}}_{O_{2}Q}$ and used descriptive statistics to demonstrate how the peak ${\dot{V}}_{O_{2}Q}$ varied according to sex, age and referral reason.

For patients tested at CUH, we divided peak ${\dot{V}}_{O_{2}Q}$ and peak ${\dot{V}}_{O_{2}}$ percent predicted into quartiles, and constructed Kaplin–Meier survival curves to show how survival probability changed as peak ${\dot{V}}_{O_{2}}$ decreased. We compared these to Kaplin–Meier survival curves for each category of the Weber functional class classification (Class A: peak ${\dot{V}}_{O_{2}}$ > 20 mL kg^−1^ min^−1^; B: ≤20 mL kg^−1^ min^−1^ but >16 mL kg^−1^ min^−1^; C: ≤16 mL kg^−1^ min^−1^ but >10 mL kg^−1^ min^−1^; D: ≤10 mL kg^−1^ min^−1^) (Weber et al., 1982). We then performed Cox proportional hazards regression analysis to investigate the association of peak ${\dot{V}}_{O_{2}Q}$, peak ${\dot{V}}_{O_{2}}$ in mL kg^−1^ min^−1^, and peak ${\dot{V}}_{O_{2}}$ percent predicted as continuous measures (per‐1‐unit increment), with all‐cause mortality. We adjusted our models for potential confounders of this association, including age, sex and BMI. We compared the prognostic ability of the three models using Harrall's C‐index. Finally, we used an interaction term to investigate the significance of any interaction between sex and peak ${\dot{V}}_{O_{2}Q}$ on mortality risk. We compared the results from the model with the interaction term to a model with the individual predictors using the likelihood ratio test. Results were considered significant if the *P*‐value was less than 0.05. Analyses were performed using Stata version 18 (StataCorp., College Station, TX, USA).

## 3 RESULTS

**FIGURE 1:**
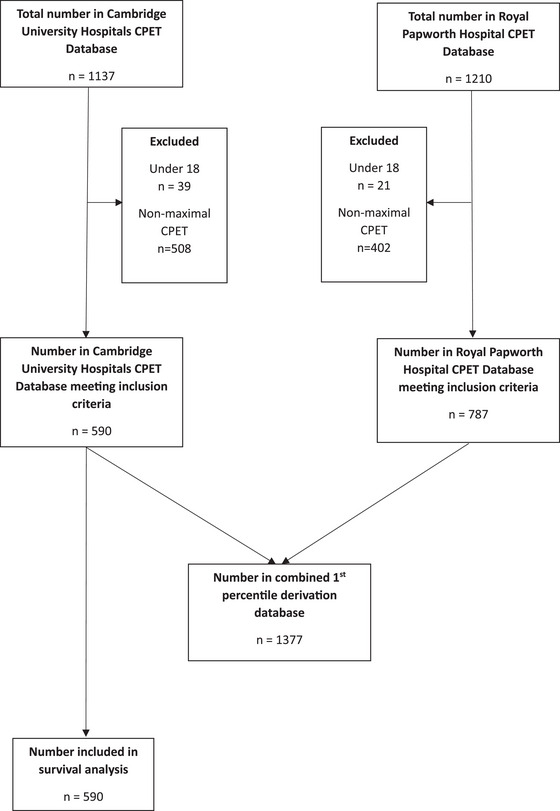
Study flow diagram.

Figure 1 shows the study flow diagram. Between the 1 January 2012 to the 31 December 2024, CPET data for 1137 individual patients were available in the CUH database. Thirty‐nine CPETs were excluded due to patients being under the age of 18, with a further 508 excluded due to being physiologically submaximal. At CUH, data from 590 patients were included in the final analyses. Mean age was 55.5 (15.4) years, 47% (279 of 590) were female, with patients referred for CPET for either unexplained shortness of breath (489 of 590, 83%) or pre‐operative assessment (101 of 590, 17%). Mean peak ${\dot{V}}_{O_{2}}$ was 23.4 (8.0) mL kg^−1^ min^−1^ or 91.8 (22.7) percent predicted (Table 1). Between 1 January 2021 and 31 December 2024, CPET data for 1210 individual patients were available in the RPH database. Twenty‐one were excluded for being under the age of 18, with a further 402 excluded due to being physiologically submaximal. For RPH, data from 787 patients were included in the final analyses. Patients from RPH were on average younger (46.1 ± 15.3 years old), with a similar proportion of females (375 of 787, 48%), but a lower peak ${\dot{V}}_{O_{2}}$ (21.0 ± 7.3 mL kg^−1^ min^−1^ or 76.1 ± 22.1 percent predicted), than those from CUH. At RPH, the predominant referral reasons were pulmonary vascular disease (240 of 787, 31%), adult congenital heart disease (222 of 787, 28%) and heart transplant assessment (214 of 787, 27%) (Table 1).

**TABLE 1:** Characteristics of patients included in the analysis.

	Cambridge University Hospitals *n* = 590	Royal Papworth Hospital *n* = 787
Age (years)	55.5 (15.4)	46.1 (15.3)
Female (*n* (%))	279 (47%)	375 (48%)
BMI (kg m^−2^)	28.2 (5.6)	27.6 (5.4)
Referral reason		
Unexplained shortness of breath (*n* (%))	489 (83%)	58 (7%)
Pre‐op assessment (*n* (%))	101 (17%)	16 (2%)
Adult congenital heart disease (*n* (%))	0 (0%)	222 (28%)
Heart transplant (*n* (%))	0 (0%)	214 (27%)
Pulmonary vascular disease (*n* (%))	0 (0%)	240 (31%)
Other	0 (0%)	37 (5%)
CPET variables		
Test duration (min)	9.8 (2.5)	8.9 (2.5)
Work rate (W)	130.0 (83.0–170.0)	105.0 (74.0–144.0)
Work rate (pp)	85.1 (31.5)	67.2 (27.1)
Peak ${\dot{V}}_{O_{2}}$ (L min^−1^)	1.9 (0.7)	1.6 (0.6)
Peak ${\dot{V}}_{O_{2}}$ (pp)	91.8 (22.7)	76.1 (22.1)
Peak ${\dot{V}}_{O_{2}}$ (mL kg^−1^ min^−1^)	23.4 (8.0)	21.0 (7.3)
d ${\dot{V}}_{O_{2}}$/dWR (mL min^−1^ W^−1^)	10.4 (9.6–11.4)	10.2 (9.3–11.1)
${\dot{V}}_{O_{2}}$ at AT (L min^−1^)	1.0 (0.4)	0.9 (0.4)
${\dot{V}}_{O_{2}}$ at AT (mL kg^−1^ min^−1^)	12.7 (4.9)	11.5 (4.4)
${\dot{V}}_{O_{2}}$ at AT/predicted peak ${\dot{V}}_{O_{2}}$	52.7 (44.1–64.0)	43.0 (35.0–55.0)
Maximum HR (bpm)	160.0 (143.0–173.0)	157.0 (135.0–172.0)
Maximum HR (pp)	95.9 (91.1–100.6)	95.0 (84.0–101.0)
Heart rate reserve (bpm)	7.0 (−1.7, 15.0)	9.0 (0.0–28.0)
End‐exercise oxygen pulse (${\dot{V}}_{O_{2}}$ /HR)	11.1 (8.7–13.9)	10.7 (8.6–13.0)
End‐exercise oxygen pulse (pp)	94.0 (75.2–116.9)	83.0 (71.0–97.0)
Cardiovascular slope (ΔHR/Δ${\dot{V}}_{O_{2}}$)	49.6 (39.5–63.8)	53.4 (39.9–70.9)
End‐exercise ${\dot{V}}_{E}$ (L min^−1^)	80.3 (29.0)	77.8 (26.3)
End‐exercise ${\dot{V}}_{E}$ (pp)	67.8 (21.4)	65.9 (22.6)
Breathing reserve at peak exercise (L min^−1^)	13.5 (2.0–34.0)	13.0 (0.0–38.0)
${\dot{V}}_{E}/{\dot{V}}_{CO_{2}}$ slope	30.0 (27.0–34.8)	33.5 (28.9–41.0)

Continuous variables presented as mean with standard deviation or median with interquartile range. Abbreviations: AT, anaerobic threshold; BMI, body mass index; bpm, beats per minute; HR, heart rate; pp, percent predicted; ${\dot{V}}_{CO_{2}}$, carbon dioxide output; ${\dot{V}}_{E}$, minute ventilation; ${\dot{V}}_{O_{2}}$, oxygen uptake.

**FIGURE 2:**
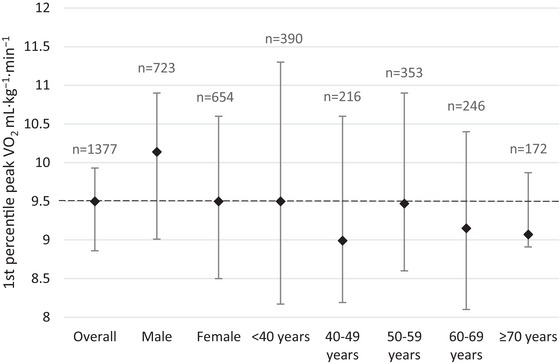
1st percentile for peak ${\dot{V}}_{O_{2}}$ stratified by sex and age. Dashed line represents the consensus 1st percentile (9.5 mL kg^−1^ min^−1^).

Data from 1377 patients were included in combined 1st percentile derivation database. Figure 2 shows the 1st percentile values overall and stratified by sex and age group. Overall, the 1st percentile value for peak ${\dot{V}}_{O_{2}}$ was 9.5 mL kg^−1^ min^−1^ (95% CI: 8.8–9.9). In males it was 10.1 mL kg^−1^ min^−1^ (95% CI: 9.0–10.9), which was similar to what was found for females, where the 1st percentile was 9.5 mL kg^−1^ min^−1^ (95% CI: 8.5–10.6). There was minimal variation in the 1st percentile value across age groups. Due to the considerable overlap of the 95% CI across sex and age groups, we chose a 1st percentile value of 9.5 mL kg^−1^ min^−1^ for peak ${\dot{V}}_{O_{2}}$, which we used to calculate the peak ${\dot{V}}_{O_{2}Q}$ for all individuals. We chose 9.5 mL kg^−1^ min^−1^ as this was the 1st percentile value for the overall population and reflected the slight difference seen between males and females. When considering the hospitals individually, the 1st percentile values were similar to the combined derivation database: CUH: 9.7 mL kg^−1^ min^−1^ (95% CI: 8.9–10.8); and RPH: 9.3 mL kg^−1^ min^−1^ (95% CI: 8.5–10.1).

Overall, the mean peak ${\dot{V}}_{O_{2}Q}$ was 2.3 (0.8), representing 2.3 turnovers from the 1st percentile value. The mean peak ${\dot{V}}_{O_{2}Q}$ was slightly higher for males than females and declined with increasing age (Table 2). At CUH, the mean peak ${\dot{V}}_{O_{2}Q}$ was 2.4 (0.7), and was lower in those undergoing pre‐operative testing, compared to those referred for unexplained shortness of breath. At RPH, the mean peak ${\dot{V}}_{O_{2}Q}$ was 2.2 (0.3), and was lowest in those referred for heart transplant assessment and highest in those referred for adult congenital heart disease (Table 2).

**TABLE 2:** Peak ${\dot{V}}_{O_{2}Q}$ overall and stratified by sex, age group and referral reason.

	Overall		Male		Female	
	*n*	Peak ${\dot{V}}_{O_{2}Q}$	*n*	Peak ${\dot{V}}_{O_{2}Q}$	*n*	Peak ${\dot{V}}_{O_{2}Q}$
Overall	1377	2.3 (0.8)	723	2.4 (0.8)	654	2.2 (0.7)
Age						
<40 years	390	2.7 (0.9)	176	2.9 (0.9)	214	2.6 (0.8)
40–49 years	216	2.4 (0.8)	104	2.6 (0.9)	112	2.3 (0.8)
50–59 years	353	2.3 (0.8)	201	2.4 (0.8)	152	2.2 (0.7)
60–69 years	246	2.0 (0.6)	144	2.2 (0.6)	102	1.9 (0.4)
≥70 years	172	1.9 (0.5)	98	2.0 (0.6)	74	1.7 (0.4)
Cambridge University Hospital						
Overall	590	2.4 (0.7)	311	2.6 (0.8)	279	2.3 (0.8)
Unexplained shortness of breath	489	2.5 (0.8)	245	2.8 (0.9)	244	2.4 (0.9)
Pre‐operative	101	2.1 (0.6)	66	2.2 (0.6)	35	1.8 (0.4)
Royal Papworth Hospital						
Overall	787	2.2 (0.8)	412	2.3 (0.8)	375	2.1 (0.7)
Unexplained shortness of breath	58	2.5 (0.8)	26	2.5 (0.7)	32	2.4 (0.8)
Pre‐op assessment	16	2.5 (0.8)	13	2.6 (0.8)	3	2.3 (0.2)
Adult congenital heart disease	222	2.6 (0.8)	94	2.9 (0.8)	128	2.4 (0.7)
Heart transplant	214	1.6 (0.4)	138	1.7 (0.4)	76	1.4 (0.3)
Pulmonary vascular disease	240	2.2 (0.6)	118	2.3 (0.6)	122	2.1 (0.6)

Peak ${\dot{V}}_{O_{2}Q}$ summarised as mean ± standard deviation (SD) for Cambridge University Hospital and Royal Papworth Hospital. No clear referral reason for 37 patients from RPH.

**FIGURE 3:**
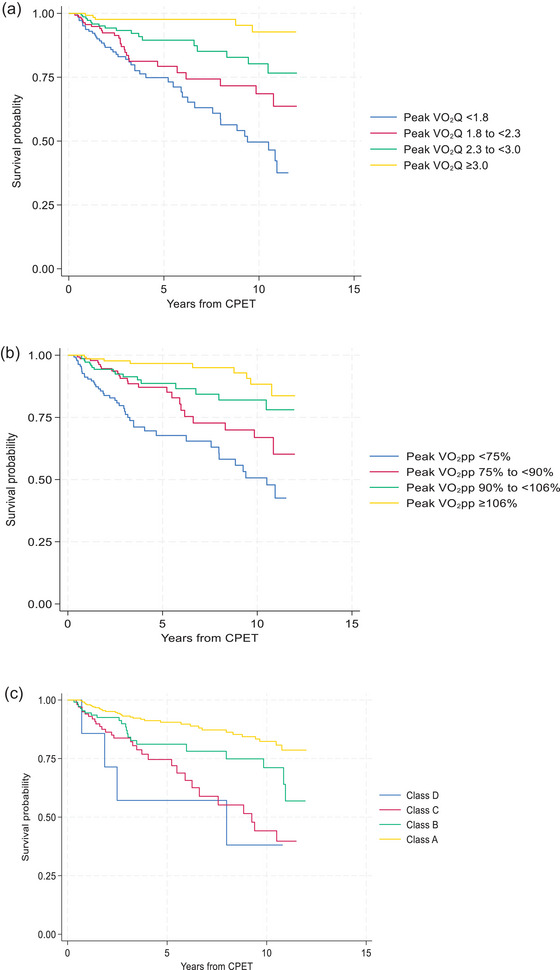
Kaplan–Meier survival curves of all‐cause mortality for quartiles of: (a) peak ${\dot{V}}_{O_{2}Q}$, (b) peak ${\dot{V}}_{O_{2}}$ percent predicted and (c) Weber functional class.

Data on all‐cause mortality were available for 590 patients at CUH. Median (IQR) follow‐up time was 3.8 (2.2–9.6) years, during which time 96 of 590 (16%) patients died. Patients who died were older, more likely to be male, to have been referred for pre‐operative assessment, and to have a lower peak ${\dot{V}}_{O_{2}Q}$ than those who were alive (Table 3). Figure 3 displays Kaplan–Meier survival curves for quartiles of peak ${\dot{V}}_{O_{2}Q}$ compared to quartiles of peak ${\dot{V}}_{O_{2}}$ percent predicted. Cumulative survival probability was similar between quartiles of peak ${\dot{V}}_{O_{2}Q}$ and peak ${\dot{V}}_{O_{2}}$ percent predicted. However, the lowest quartile for peak ${\dot{V}}_{O_{2}Q}$ had a steeper slope, with a higher event rate, while the highest quartile had a shallower slope and lower event rate, compared to quartiles of peak ${\dot{V}}_{O_{2}}$ percent predicted. There was also greater separation between the slopes for each quartile of peak ${\dot{V}}_{O_{2}Q}$ compared to peak ${\dot{V}}_{O_{2}}$ percent predicted. In comparison, Weber functional class classifications performed poorly, with significant overlap in cumulative survival probability across classifications. The absolute risk of death among those in the lowest quartile for peak ${\dot{V}}_{O_{2}Q}$ (<1.8) was 30% (45 of 148), compared to 3% (5 of 147) in the highest quartile (≥3.0). Those in the highest quartile had an 87% lower risk of all‐cause mortality compared to those in the lowest quartile (Table 4). Compared to those in the higher quartiles, those in the lowest quartile for peak ${\dot{V}}_{O_{2}Q}$ were older, with a higher BMI, lower anaerobic threshold, lower oxygen pulse, steeper heart rate slope and higher ${\dot{V}}_{E}/{\dot{V}}_{CO_{2}}$ (Table 5).

**TABLE 3:** Characteristics of those included in the survival analysis at Cambridge University Hospital stratified by mortality status.

	Alive (*n* = 494)	Deceased (*n* = 96)	*P*
Age (years)	53.2 (15.4)	67.2 (9.4)	<0.0001
Female (*n* (%))	250 (51%)	28 (29%)	<0.0001
BMI (kg m^−2^)	28.1 (5.7)	28.3 (5.0)	0.756
Pre‐operative (*n* (%))	51 (10%)	50 (52%)	<0.0001
CPET variables			
Test duration (min)	9.9 (2.4)	9.4 (2.8)	0.112
Work rate (W)	135.0 (87–174.0)	88.0 (67.0–135.0)	<0.0001
Work rate (pp)	86.8 (29.3)	71.4 (39.7)	0.006
Peak ${\dot{V}}_{O_{2}}$ (L min^−1^)	1.9 (0.7)	1.5 (0.5)	<0.0001
Peak ${\dot{V}}_{O_{2}}$ (pp)	94.2 (22.5)	79.5 (19.3)	<0.0001
Peak ${\dot{V}}_{O_{2}}$ (mL kg^−1^ min^−1^)	24.3 (8.2)	18.9 (5.5)	<0.0001
Peak ${\dot{V}}_{O_{2}Q}$	2.6 (0.9)	2.0 (0.6)	<0.0001
d ${\dot{V}}_{O_{2}}$/dWR (mL min^−1^ W^−1^)	10.6 (9.6–11.4)	10.0 (9.1–11.4)	0.086
${\dot{V}}_{O_{2}}$ at AT (L min^−1^)	1.0 (0.4)	0.9 (0.3)	<0.0001
${\dot{V}}_{O_{2}}$ at AT (mL kg^−1^ min^−1^)	13.1 (5.0)	10.9 (4.0)	<0.0001
Maximum HR (bpm)	163.0 (148.0–174.0)	145.0 (132.5–161.0)	<0.0001
Maximum HR (pp)	95.8 (91.4–100.5)	95.9 (88.2–103.1)	0.849
Heart rate reserve (bpm)	7.0 (−1.0, 15.0)	6.6 (−5.0, 17.5)	0.593
End‐exercise oxygen pulse (${\dot{V}}_{O_{2}}$/HR)	11.2 (9.1–14.1)	9.6 (8.0–12.2)	<0.0001
End‐exercise oxygen pulse (pp)	93.3 (75.5–116.5)	99.4 (73.2–118.8)	0.219
Cardiovascular slope (ΔHR/Δ${\dot{V}}_{O_{2}}$)	49.3 (39.0–63.8)	51.6 (40.0–64.4)	0.619
End‐exercise ${\dot{V}}_{E}$ (L min^−1^)	82.5 (29.0)	69.0 (26.0)	<0.0001
Breathing reserve at peak exercise (L min^−1^)	14.3 (2.9–34.6)	11.4 (−1.3, 29.6)	0.160
${\dot{V}}_{E}/{\dot{V}}_{CO_{2}}$ slope	29.9 (26.9–34.4)	31.4 (27.1–26.1)	0.177

Continuous variables presented as mean with standard deviation or median with interquartile range. Binary data compared using chi‐squared test. Continuous data compared using independent samples *t*‐test for parametric data and Mann‐Whitney *U*‐test for non‐parametric data. Abbreviations: AT, anaerobic threshold; BMI, body mass index; bpm, beats per minute; HR, heart rate; pp, percent predicted; ${\dot{V}}_{CO_{2}}$, carbon dioxide output; ${\dot{V}}_{E}$, minute ventilation; ${\dot{V}}_{O_{2}}$, oxygen uptake.

**TABLE 4:** Association of peak ${\dot{V}}_{O_{2}Q}$ quartiles with all‐cause mortality.

Peak ${\dot{V}}_{O_{2}Q}$ quartiles	*n*	Deceased *n* (%)	HR (95% CI)	*P*
<1.8	148	45 (30%)	Ref	Ref
1.8 to <2.3	147	28 (19%)	0.54 (0.33–0.89)	0.015
2.3 to <3.0	148	18 (12%)	0.38 (0.20–0.70)	0.002
≥3.0	147	5 (3%)	0.13 (0.05–0.38)	<0.0001

Abbreviations: HR, hazard ratio; ${\dot{V}}_{O_{2}}$, oxygen uptake; ref, reference group.

**TABLE 5:** Characteristics of those included in the survival analysis stratified by peak ${\dot{V}}_{O_{2}Q}$ quartile.

	Peak ${\dot{V}}_{O_{2}Q}$ <1.8 (*n* = 148)	Peak ${\dot{V}}_{O_{2}Q}$ 1.8 to <2.3 (*n* = 147)	Peak ${\dot{V}}_{O_{2}Q}$ 2.3 to <3.0 (*n* = 148)	Peak ${\dot{V}}_{O_{2}Q}$ ≥3.0 (*n* = 147)
Age (years)	66.2 (11.1)	59.7 (12.9)	52.7 (14.0)	43.2 (13.4)
Female (*n* (%))	95 (64%)	78 (53%)	52 (35%)	53 (36%)
BMI (kg m^−2^)	30.9 (6.3)	28.8 (6.1)	27.6 (4.3)	25.2 (3.6)
Pre‐operative (*n* (%))	39 (26%)	32 (22%)	23 (16%)	7 (5%)
CPET variables				
Test duration (min)	7.5 (2.1)	9.6 (2.0)	10.6 (1.8)	11.5 (2.0)
Work rate (W)	67 (51–88)	116 (83–133)	147 (129–173)	195 (158–240)
Work rate (pp)	59.0 (23.8)	78.7 (23.0)	93.8 (28.4)	108.9 (25.4)
Peak ${\dot{V}}_{O_{2}}$ (L min^−1^)	1.2 (0.3)	1.6 (0.4)	2.0 (0.4)	2.6 (0.6)
Peak ${\dot{V}}_{O_{2}}$ (pp)	70.0 (12.1)	85.6 (13.0)	96.3 (14.6)	115.1 (21.0)
Peak ${\dot{V}}_{O_{2}}$ (mL kg^−1^ min^−1^)	14.4 (2.0)	19.7 (1.4)	24.9 (2.0)	34.4 (5.3)
Peak ${\dot{V}}_{O_{2}Q}$	1.5 (0.2)	2.1 (0.1)	2.6 (0.2)	3.6 (0.6)
d ${\dot{V}}_{O_{2}}$/dWR (mL min^−1^ W^−1^)	9.9 (9.0–11.7)	10.4 (9.5–11.4)	10.3 (9.6–11.0)	10.7 (9.9–10.7)
${\dot{V}}_{O_{2}}$ at AT (mL kg^−1^ min^−1^)	8.4 (3.7)	11.4 (2.8)	13.3 (3.0)	17.8 (4.3)
Maximum HR (bpm)	137 (122–155)	156 (142–167)	165 (155–174)	173 (162–184)
Maximum HR (pp)	91.5 (78.5–96.7)	95.2 (90.2–101.3)	97.6 (93.9–101.7)	97.6 (93.56–101.3)
Heart rate reserve (bpm)	10.3 (5.0–20.1)	8.0 (−2.0–16.0)	4.0 (−2.6–11.0)	4.0 (−2.2 to 11.0)
End‐exercise oxygen pulse (${\dot{V}}_{O_{2}}$/HR)	8.4 (7.0–10.3)	10.1 (8.0–12.1)	12.4 (10.2–13.8)	14.7 (11.9–17.9)
End‐exercise oxygen pulse (pp)	79.5 (63.0–103.6)	84.8 (73.2–102.9)	93.9 (77.8–105.1)	114.3 (97.0–131.5)
Cardiovascular slope (ΔHR/Δ${\dot{V}}_{O_{2}}$)	54.3 (43.5‐73.7)	53.0 (41.9–68.7)	47.1 (39.8–59.4)	42.9 (34.0–53.5)
End‐exercise ${\dot{V}}_{E}$ (L min^−1^)	55.0 (17.6)	73.2 (21.2)	87.0 (20.9)	105.5 (27.7)
Breathing reserve at peak exercise (L min^−1^)	12.1 (4.3‐31.0)	12.8 (6.0–27.8)	17.7 (4.9–37.0)	18.3 (−1.1 to 38.3)
${\dot{V}}_{E}/{\dot{V}}_{CO_{2}}$ Slope	35.8 (30.2–50.5)	31.7 (29.0–36.0)	28.8 (26.1–31.7)	27.0 (24.8–29.8)

Continuous variables presented as mean with standard deviation or median with interquartile range. Abbreviations: AT, anaerobic threshold; BMI, body mass index; bpm, beats per minute; HR, heart rate; pp, percent predicted; ${\dot{V}}_{CO_{2}}$, carbon dioxide output; ${\dot{V}}_{E}$, minute ventilation; ${\dot{V}}_{O_{2}}$, oxygen uptake.

Table 6 shows the association between a 1‐unit increase in peak ${\dot{V}}_{O_{2}Q}$, peak ${\dot{V}}_{O_{2}}$ mL kg^−1^ min^−1^, and peak ${\dot{V}}_{O_{2}}$ percent predicted with all‐cause mortality. In the adjusted models, a 1‐unit increase in peak ${\dot{V}}_{O_{2}Q}$ was associated with a 60% reduction in all‐cause mortality risk (HR: 0.40, 95% CI: 0.27–0.61), a 1‐unit increase in peak ${\dot{V}}_{O_{2}}$ mL kg^−1^ min^−1^ was associated with a 9% reduction (HR: 0.91, 95% CI: 0.87–0.95), and a 1‐unit increase in percent predicted peak ${\dot{V}}_{O_{2}}$ was associated with a 2% reduction (HR: 0.98, 95% CI: 0.97–0.99). In standardised units (per 1‐SD increase), peak ${\dot{V}}_{O_{2}Q}$ (HR: 0.46, 95% CI: 0.33–0.65, *P *< 0.0001), peak ${\dot{V}}_{O_{2}}$ mL kg^−1^ min^−1^ (HR: 0.49, 95% CI: 0.34‐0.67, *P *< 0.0001), and peak ${\dot{V}}_{O_{2}}$ percent predicted (HR: 0.58, 95% CI: 0.46‐0.77, *P *< 0.0001) were similarly associated with mortality. Harrell's *C*‐index was 0.79, 0.78 and 0.77, respectively. There was no significant interaction between sex and peak ${\dot{V}}_{O_{2}Q}$ on risk of mortality (*P*‐value for likelihood ratio = 0.373).

**TABLE 6:** Association of peak ${\dot{V}}_{O_{2}Q}$, peak ${\dot{V}}_{O_{2}}$ mL kg^−1^ min^−1^, and peak ${\dot{V}}_{O_{2}}$ percent predicted with all‐cause mortality.

	*n*	Deceased (*n* (%))	HR (95% CI)	*P*	Harrell's *C*
Unadjusted model					
Peak ${\dot{V}}_{O_{2}Q}$	590	96 (16%)	0.36 (0.28–0.59)	<0.0001	0.70
Peak ${\dot{V}}_{O_{2}}$ mL kg^−1^ min^−1^	590	96 (16%)	0.90 (0.87–0.93)	<0.0001	0.68
Peak ${\dot{V}}_{O_{2}}$ pp	590	96 (16%)	0.97 (0.96–0.98)	<0.0001	0.70
Adjusted model					
Peak ${\dot{V}}_{O_{2}Q}$	590	96 (16%)	0.40 (0.27–0.61)	<0.0001	0.79
Peak ${\dot{V}}_{O_{2}}$ mL kg^−1^ min^−1^	590	96 (16%)	0.91 (0.87–0.95)	<0.0001	0.78
Peak ${\dot{V}}_{O_{2}}$ pp	590	96 (16%)	0.98 (0.97–0.99)	<0.0001	0.77

Results interpreted as per 1‐unit increment in independent predictor. Adjusted model accounts for age, sex, and BMI. Abbreviations: HR, hazard ratio; pp, percent predicted; ${\dot{V}}_{O_{2}}$, oxygen uptake.

## 4 DISCUSSION

We have shown that the 1st percentile for peak ${\dot{V}}_{O_{2}}$ was consistent across sex and age groups. We have demonstrated how the derived peak ${\dot{V}}_{O_{2}Q}$ varies with disease and shown that it predicts all‐cause mortality at least as well as peak ${\dot{V}}_{O_{2}}$ percent predicted, without the need for reference equations.

Using data from patients performing CPET in two hospital settings, we found that the 1st percentile value for peak ${\dot{V}}_{O_{2}}$ was approximately 9.5 mL kg^−1^ min^−1^ and was consistent across sex and age groups, and replicated in both hospital settings. Given the wide variation in normal values for peak ${\dot{V}}_{O_{2}}$ seen in the populations used to derive reference equations (Koch et al., 2009; Myers et al., 2017; Wasserman et al., 1987), this could be considered surprising. However, as peak ${\dot{V}}_{O_{2}}$ expressed in mL kg^−1^ min^−1^ accounts for the influence of bodyweight, it is perhaps logical that there is a consistent minimal value required to sustain life. In our study, patients closest to this value were those undergoing heart transplant assessment at RPH. These patients had a mean peak ${\dot{V}}_{O_{2}Q}$ of 1.6, representing being 1.6 turnovers away from the 1st percentile value. In the most recent International Society for Heart and Lung Transplantation guidelines for the evaluation and care of cardiac transplant candidates, a peak ${\dot{V}}_{O_{2}}$ ≤14.0 mL kg^−1^ min^−1^ for those not on beta‐blockers was recommended to support listing for transplant (Peled et al., 2024). This value is equivalent to a peak ${\dot{V}}_{O_{2}Q}$ of 1.5, similar to the mean peak ${\dot{V}}_{O_{2}Q}$ shown for heart transplant assessment patients in our study. Interestingly, the first studies to inadvertently demonstrate the potential of a minimally survivable lower boundary for peak ${\dot{V}}_{O_{2}}$ were the seminal work by Mancini et al. (1991) and Szlachcic et al. (1985). They demonstrated in patients undergoing heart transplant assessment, that there was a small group of patients with a peak ${\dot{V}}_{O_{2}}$ less than 10 mL kg^−1^ min^−1^, where outcomes were significantly worse, with a 1‐year mortality rate of over 70%. Similarly, Weber et al. (1982) demonstrated that heart failure patients could be classified into different functional classifications based on peak ${\dot{V}}_{O_{2}}$, and that the patients with the most severe functional limitation had a peak ${\dot{V}}_{O_{2}}$ <10 mL kg^−1^ min^−1^. This supports our finding, that the 1st percentile value in a patient population is approximately 9.5 mL kg^−1^ min^−1^, describing patients who, while still ambulatory, are the most clinically unwell.

The association between cardiorespiratory fitness and all‐cause mortality is well described in the literature (Cai et al., 2023; Gonzales et al., 2021; Mancini et al., 1991; Mandsager et al., 2018; Steell et al., 2019; Szlachcic et al., 1985), with a low peak ${\dot{V}}_{O_{2}}$ indicative of an impairment in the cardiovascular respiratory, and/or metabolic systems. In the present study, we found that peak ${\dot{V}}_{O_{2}Q}$ was strongly associated with all‐cause mortality. When comparing survival curves for quartiles of peak ${\dot{V}}_{O_{2}Q}$, we found that in those in the lowest quartile, with a peak ${\dot{V}}_{O_{2}Q}$ less than 1.8, the absolute risk of mortality over a median of 3.8 years, was 30%. The association for quartiles of peak ${\dot{V}}_{O_{2}}$ percent predicted was similar, but graphically did not seem to discriminate survival as well as peak ${\dot{V}}_{O_{2}Q}$, with survival probability being better for those in the highest quartile of peak ${\dot{V}}_{O_{2}Q}$ (shallower curve) and worse for those in the lowest quartile (steeper curve). This may represent the limitations of reference equations, particularly in older age groups, as some in the highest quartile for peak ${\dot{V}}_{O_{2}}$ percent predicted, who subsequently died, were classified in the second lowest quartile for peak ${\dot{V}}_{O_{2}Q}$. These were all patients over the age of 70 years, who were likely wrongly classified as having a peak ${\dot{V}}_{O_{2}}$ above 106% predicted, when in reality, they were significantly closer to the 1st percentile value. When using the Weber functional class classifications, we found that relatively few deaths occurred in classes B and D, compared to A and C, suggesting that in comparison to quartiles of peak ${\dot{V}}_{O_{2}Q}$, the Weber peak ${\dot{V}}_{O_{2}}$ cut‐offs do not discriminate those with the lowest and highest mortality risk as effectively. We propose that a peak ${\dot{V}}_{O_{2}Q}$ of <1.8 could be used to define functional limitation, not only due to the increased mortality risk, but also because these patients had an abnormal physiological response to exercise when using traditional definitions, for example peak ${\dot{V}}_{O_{2}}$ and oxygen pulse <80% predicted, anaerobic threshold <40% of peak predicted ${\dot{V}}_{O_{2}}$, and ${\dot{V}}_{E}/{\dot{V}}_{CO_{2}}$ >30 (Glaab & Taube, 2022).

After adjustment for confounding, we found that per 1‐unit increase in peak ${\dot{V}}_{O_{2}Q}$, reflecting being 1 turnover further from the 1st percentile, risk of all‐cause mortality reduced by 60%. This relationship did not vary by sex, supporting the use of a universal 1st percentile comparator. After standardising the units, the association of peak ${\dot{V}}_{O_{2}}$ mL kg^−1^ min^−1^ and peak ${\dot{V}}_{O_{2}}$ percent predicted with all‐cause mortality was similar. While the peak ${\dot{V}}_{O_{2}Q}$ did not dramatically improve mortality prediction, it has the advantage that it does not require refence equations, and unlike when raw units are used, for example mL kg^−1^ min^−1^, cardiorespiratory fitness estimates are still being compared to a universal standard, which is conceptually easier to understand, especially for those without specialist training. This could be particularly useful in pre‐operative assessments, where a minimum survivable level of cardiorespiratory fitness may be a more appropriate comparator than an ideal predicted level, although, more research in specific patient groups is needed to fully understand the utility of peak ${\dot{V}}_{O_{2}Q}$.

The strengths of our study include the large sample of physiologically maximal CPETs from patients covering a wide spectrum of referral reasons. Furthermore, CPETs were performed to strict standards, by highly trained respiratory physiologists, which ensures the quality of data collected. Limitations of our study include the exclusion of a large proportion of physiologically submaximal CPETs, which reduced the sample size for stratified analyses. Furthermore, we did not have access to survival data for patients at RPH, meaning further work is needed to explore the association of the peak ${\dot{V}}_{O_{2}Q}$ with mortality and other clinical outcomes in specific diseases. In addition, as CPET reference equations do not consider race, we did not have accurate information on this in our databases. From previous research, our patient cohorts were approximately 95% White European, meaning it was not possible for us to explore whether the 1st percentile value varied according to racial group. However, it is generally accepted that variation in cardiorespiratory fitness estimates across races is significantly influenced by lifestyle and social determinants, not purely genetic differences (Ceaser & Hunter, 2015; Nightingale et al., 2016). Finally, despite drawing on data from two separate hospital samples, the 1st percentile value for peak ${\dot{V}}_{O_{2}Q}$ would benefit from external validation in similar cohorts, especially in comparison to CPETs performed on a treadmill, where estimates of peak ${\dot{V}}_{O_{2}}$ can be higher (Loftin et al., 2004).

In conclusion, we have shown that the 1st percentile for peak ${\dot{V}}_{O_{2}}$ was consistent across sex and age groups, and when used to generate the peak ${\dot{V}}_{O_{2}Q}$, was strongly associated with all‐cause mortality. We propose the peak ${\dot{V}}_{O_{2}Q}$ as an alternative means to interpret cardiorespiratory fitness estimates, without the need for reference equations. Future research should focus on the prognostic ability of the peak ${\dot{V}}_{O_{2}Q}$ in different patient populations.

## AUTHOR CONTRIBUTIONS

Ben Knox‐Brown and Karl P. Sylvester conceived the study. Chris Harding and Joshua Barnes constructed the databases at CUH and RPH. Ben Knox‐Brown performed data analysis and drafted the first version of the manuscript with input from Karl P. Sylvester and Jonathan Fuld. Jonathan Fuld, Karl P. Sylvester, Chris Harding, and Joshua Barnes contributed critical intellectual content to further revisions of the manuscript. Ben Knox‐Brown is the guarantor of the data. All authors have read and approved the final version of this manuscript and agree to be accountable for all aspects of the work in ensuring that questions related to the accuracy or integrity of any part of the work are appropriately investigated and resolved. All persons designated as authors qualify for authorship, and all those who qualify for authorship are listed.

## CONFLICT OF INTEREST

K.S. has received consultation fees from ndd medical unrelated to this work. J.B. has received consultation fees from Vitalograph unrelated to this work. All other authors have no conflicts of interest to disclose.

## FUNDING INFORMATION

None.

## Data Availability

This study uses routinely collected health care data from NHS patients in the United Kingdom, as such, data is not freely available. However, data can be made available upon reasonable request by individuals employed or in receipt of an honorary contact at either Royal Papworth or Cambridge University Hospitals.
